# The predictive ability of indirect genetic models is reduced when culled animals are omitted from the data

**DOI:** 10.1186/s12711-020-0527-x

**Published:** 2020-02-10

**Authors:** Birgitte Ask, Ole F. Christensen, Marzieh Heidaritabar, Per Madsen, Hanne M. Nielsen

**Affiliations:** 1 SEGES, Danish Pig Research Centre, Danish Agriculture & Food Council F.m.b.A., Axelborg, Axeltorv 3, 1609 Copenhagen V, Denmark; 2 Department of Molecular Biology and Genetics-Center for Quantitative Genetics and Genomics, Blichers Allé 20, 8830 Tjele, Denmark; 3 Present Address: Department of Molecular Biology and Genetics-Center for Quantitative Genetics and Genomics, Blichers Allé 20, 8830 Tjele, Denmark

## Abstract

**Background:**

Physical removal of individuals from groups causes reductions in group sizes and changes in group composition, which may affect the predictive ability of estimates of indirect genetic effects of animals on phenotypes of group mates. We hypothesized that including indirect genetic effects of culled animals and of animals without phenotypes in the analysis affects estimates of genetic parameters, improves predictive ability, and reduces bias of predicted breeding values. We tested this by applying different editing procedures, i.e. omission of individuals or groups from the data, and genetic models, i.e. a classical and an indirect genetic model (IGM) without or with weighting of indirect genetic effects based on the relative proportion of time spent in the pen or space allowance. Data consisted of average daily gain for 123,567 pigs in 11,111 groups, from which 3% of individuals in 25% of groups were prematurely removed from the group.

**Results:**

The estimate of total heritability was higher (0.29 to 0.34) than that of direct heritability (0.23 to 0.25) regardless of the editing procedures and IGM used. Omission of individuals or groups from the data reduced the predictive ability of estimates of indirect genetic effects by 8 to 46%, and the predictive ability of estimates of the combined direct and indirect genetic effects by up to 4%. Omission of full groups introduced bias in predicted breeding values. Weighting of indirect genetic effects reduced the predictive ability of their estimates by at least 19% and of the estimates of the combined direct and indirect genetic effects by 1%.

**Conclusions:**

We identified significant indirect genetic effects for growth in pigs. Culled animals should neither be removed from the data nor accounted for by weighting their indirect genetic effects in the model based on the relative proportion of time spent in the pen or space allowance, because it will reduce predictive ability and increase bias of predicted breeding values. Information on culled animals is important for prediction of indirect genetic effects and must be accounted for in IGM analyses by including fixed regressions based on relative time spent within the pen or relative space allowance.

## Background

An indirect genetic model (IGM) for estimation of breeding values includes both direct and indirect genetic effects of animals. It has been suggested that estimated breeding values from such a model can be used to select for social behavior without the need for expensive and tedious recording of complex behavioral traits, such as tail biting in pigs. Ellen et al. [1] showed that selection on estimates of indirect genetic effects led to improvement in survival of layers housed in groups. Several studies have also identified significant indirect genetic effects for growth for pigs housed in groups [2, 3]. Previously reported predictive ability of combined direct and indirect genetic effects has not been consistently higher than that of direct genetic effects alone [3, 4]. A reason for this may be that for many species, e.g. fish, layer chickens, or pigs, groups of animals are typically subject to premature physical removal of one or several animals from the group (from here on collectively referred to as culling). Specifically, in pig breeding, individual pigs can be removed from pens during performance testing due to e.g. death, disease, leg problems, tail biting, or sale. Culling of animals from groups reduces group size and changes group composition across the test period, which may pose a challenge in IGM if it is ignored.

There are several reasons why culled animals should not be ignored in IGM. First, the phenotypes of all group members are affected when an individual is culled, because the phenotype of an individual is the result of its direct genetic effect plus the sum of the indirect genetic effects of its group mates [5]. Second, culling affects the covariance between phenotype and total breeding value, because it is a function of not only direct and indirect genetic (co-)variances, but also of group size and additive genetic relatedness among group mates [5]. These parameters are unintentionally modified when animals are culled from the group, which may affect predictive ability of estimated genetic effects [3, 4]. In some previous studies on indirect genetic effects, animals that were culled, or even complete groups that were culled, were omitted from the dataset before analysis [6, 7]. However, if the culling of animals is non-random, removal of these animals from analysis could bias, in particular, estimates of the genetic (co-)variance between direct and indirect genetic effects from the IGM [8–10]. At least part of the culled or removed animals are likely to be non-random with regards to ADG as e.g. animals that are sold would typically be sold based partly on breeding values and partly on body weight. In this case, the missing data process cannot be ignored [11]. Thus, the culling of animals may need to be accounted for in the IGM to avoid biases and improve predictive ability.

Another challenge associated with culling of animals is that differences in time spent in the group and changes in space allowance per animal may also affect the indirect genetic effect of a given animal. Cantet and Cappa [12] suggested a method to weigh indirect genetic effects based on the amount of time spent in a group. Bunter et al. [7] weighted indirect genetic effects on litter size in sows based on space allowance. However, to our knowledge, the model of Cantet and Cappa [12] has never been tested on real data and the model by Bunter et al. [7] has never been tested on growth data in pigs. We propose to apply models similar to those suggested by Cantet and Cappa [12] and Bunter et al. [7] to growth data in pigs to account for culling of animals instead of omitting them from the dataset. Our aim was to investigate the effect of accounting for culling of animals on genetic parameters and on predictive ability and bias of estimated genetic effects, both through the data editing procedure and through weighting of indirect genetic effects in an IGM.

## Methods

### Ethics statement

The data used were part of routine recording of pigs in performance tests in nucleus herds of the DanBred pig breeding program. Management and welfare procedures were in accordance with Danish national standards and the records obtained in these tests, e.g. weight, require no ethical approval.

### Pigs and housing

Data were from performance-tested DanBred Landrace pigs in 18 nucleus breeding herds within the period from January 2014 to May 2017. The age of the pigs at the start of the performance test was on average 82 days [standard deviation (sd) = 8.2] and ranged from 42 to 156 days. The average starting weight was 30 kg (sd = 1.9), ranging from 20 to 40 kg, and the average weight at the end of the performance test was 94 kg (sd = 9.0), ranging from 45 to 140 kg. The number of test days per pig was on average 63 days (sd = 6.9), ranging from 34 to 93 days. Average daily gain (ADG) was calculated as:

$$ADG \left( {g/day} \right) = \frac{{\left( {weight_{end} - weight_{start} } \right) \times 1000}}{days\,in\,performance\,test},$$

where $weight_{end}$ and $weight_{start}$ were the weights in kg at the end and start of the performance test, respectively. Groups consisted of either boars or gilts. Group sizes at the start of the performance test ranged from 8 to 15 pigs per pen and the space allowance per pig was between 0.75 and 1.0 m^2^ at the start of the performance test. If a pig left the pen because of death, illness, or sale, the date and reason for leaving the pen were recorded. Pigs were neither allowed to re-enter their pen after having been culled, nor to move to any other pen that was part of the performance test. During the test, feeding was ad libitum with dry feed, which at a minimum fulfills the Danish norm requirements given by SEGES [13].

### Data and editing procedure scenarios

Descriptive statistics after editing (described below), including numbers of animals, litters, and groups, as well as group sizes, percentage of missing phenotypes, mean ADG, and average additive genetic relationship for six datasets that represent different scenarios of editing procedures regarding culling of pigs or missing phenotypes are in Table 1.

**Table 1:** Descriptive statistics of the datasets in the reference scenario *full* and the five alternative scenarios

	*full*	*noPhen*	*testdays*	*noPhenGrp*	*noPhenGrp15*	*predPhen*
Number of animals	123,567	118,147	122,974	81,825	115,869	123,567
Number of sires	676	676	676	669	675	676
Number of dams	12,293	12,249	12,288	11,678	12,226	12,293
Number of litters	16,681	16,613	16,674	15,739	16,587	16,681
Number of groups	11,111	11,111	11,111	7473	10,387	11,111
% males	41.3	41.2	41.3	39.0	42.0	41.3
% male groups	40.1	40.1	40.1	38.0	40.9	40.1
Group size; mean (min–max)	11.6 (8–15)	11.1 (2–15)	11.5 (5–15)	11.4 (8–15)	11.6 (8–15)	11.6 (8–15)
Space allowance ($m^{2} /pig$); mean (min–max)	0.88 (0.76–1.00)	0.92 (0.76–6.10)	0.89 (0.76–1.83)	0.89 (0.76–1.00)	0.88 (0.76–1.00)	0.88 (0.76–1.00)
% pigs without phenotype (or culled)	4.4	0	3.9	0	3.1	0
% groups with missing phenotypes (or culled)	32.7	0	29.7	0	28.1	0
% pigs culled	3.2	0	1.2	0	0.9	0
% groups with culled pigs	24.8	0	9.4	0	8.3	0
Additive genetic relationship within pens; mean (min–max)	0.17 (0.07–0.55)	0.17 (0.07–0.55)	0.17 (0.07–0.55)	0.17 (0.07–0.49)	0.17 (0.07–0.55)	0.17 (0.07–0.55)
ADG; mean g/d ± sd (min–max)	1017 ± 126 (377–1698)	1017 ± 126 (377–1698)	1017 ± 126 (377–1698)	1020 ± 124 (382–1698)	1017 ± 126 (377–1698)	1017 ± 125 (377–1698)
$twg_{ij}$: proportion of time that pigs spent with each other within groups; mean (min–max)	0.987 (0.476–1.000)	N/A	0.991 (0.476–1.000)	N/A	0.992 (0.870–1.000)	0.987 (0.476–1.000)
$sbg_{grp}$: relative space allowance among groups; mean (min–max)	1.011 (0.239–1.204)	N/A	1.012 (0.239–1.204)	N/A	1.025 (0.802–1.204)	1.011 (0.239–1.204)
$twsbg_{ij}$: the product of $twg_{ij}$ and $sbg_{grp}$; mean (min–max)	0.999 (0.114–1.204)	N/A	1.004 (0.114–1.204)	N/A	1.017 (0.720–1.204)	0.999 (0.114–1.204)

Alternative scenarios were: *noPhen* (individually culled pigs or pigs without phenotypes were omitted), *testdays* (pigs culled within the first 28% of test days were omitted), *noPhenGrp* (complete groups with culled pigs or pigs with missing phenotypes omitted), *noPhenGrp15* (groups with more than 15% culled pigs or missing phenotypes were omitted), and *predPhen* (predicted breeding values assigned to pigs without phenotypes including culled pigs)

Groups with the following characteristics were omitted from all datasets:

- Group sizes smaller than 8 or greater than 15 at the start of the performance test, because they were considered too rare (68 groups omitted).
- Groups with one or more pigs having pedigree errors (105 groups omitted).
- Groups with a smaller group size at the start than the pen size prescribed (to ensure similar space allowance at the beginning of the test) or with any pigs unaccounted for at the end of the performance test, i.e. physically not in the pen, but not recorded as having been culled (285 groups omitted).
- Group sizes that occurred infrequently, either within a herd (less than 10 groups per herd with a given group size; 37 groups omitted), or within a sex (less than 10 groups per sex with a given group size; 20 groups omitted).

The six datasets consisted of a reference scenario (*full*) without any further edits based on culling of pigs from the groups or missing phenotypes and five alternative editing procedures based on culling of pigs from groups or missing phenotypes (*noPhen*, *testdays*, *noPhenGrp*, *noPhenGrp15*, and *predPhen*), as described below. Culling of pigs was due to e.g. disease, leg problems, tail bites, or sale and missing phenotypes (for pigs that were still in the pen) were due to recording errors (i.e. missing phenotypes). The five datasets for the alternative scenarios were subsets of the dataset for the *full* reference scenario.

In the *full* scenario, there was no data editing based on culled pigs. Thus, the data for this scenario included all pigs that were in the pens at the start of the performance test, i.e. including pigs that died and that were culled, and pigs that did not have phenotypic records although they were present at the end of the performance test. In total, 1.2% of pigs did not receive a phenotype although they were present at the end of the performance test, thereby affecting 7.9% of groups. The dataset for the *full* scenario consisted of 123,567 pigs in 11,111 groups, with group size ranging from 8 to 15 at the start of the performance test. In total, 4.4% of the pigs and 32.7% of the groups had missing phenotypes. The mean ADG was 1017 g/d, and the additive genetic relationship within pens based on six generations was on average 0.17, with the first and third quantiles being 0.14 and 0.18, respectively (Table 1).

In the *noPhen* scenario, pigs without phenotypes were omitted from the dataset regardless of when or whether they were in reality physically removed from the group. This means that none of the pigs and none of the groups in this dataset had missing phenotypes. Group size equaled the number of pigs that received a phenotype within that group and ranged from 2 to 15. The mean ADG and the average additive genetic relationship within pens were equivalent to those of the *full* scenario (Table 1).

In the *testdays* scenario, pigs that were culled from the group during the first 28% of days in the performance test were omitted. We chose this 28% value because it reduced the percentage of groups with culled pigs to less than 10% (compared to ~ 25% in the *full* scenario). Group size was defined as the number of pigs in the group when 28% of the performance test days had passed. Group size ranged from 5 to 15, and 3.9% of the pigs and 29.7% of the groups had missing phenotypes. The mean ADG and the average additive genetic relationship within pens were equivalent to those of the *full* scenario (Table 1).

In the *noPhenGrp* scenario, entire groups with culled pigs or with pigs with missing phenotypes were omitted. Group size equaled that at the start of the performance test, as in the scenario *full*. The mean ADG was 1020 g/day and the additive genetic relationship within pens was on average 0.17, with the first and third quantiles being 0.14 and 0.19, respectively (Table 1).

In the *noPhenGrp15* scenario, an entire group was omitted when the proportion of missing phenotypes in the group was higher than 15%. We chose this 15% value because it reduced the percentage of groups with culled pigs or missing phenotypes to less than 10%. Group size equaled that at the start of the performance test, as in the scenario *full*. In total, 3.1% of the pigs and 28.1% of the groups still had missing phenotypes. The mean ADG and the average additive genetic relationship within pens were equivalent to those in the *full* scenario (Table 1).

In the *predPhen* scenario, no pigs or groups were omitted, i.e. the number of pigs and groups equaled those in the *full* scenario. Instead, predicted direct breeding values, $\hat{a}_{Di} = ebv_{D,i}$, which were based on the classical genetic model (see next section) applied to the *full* dataset, were assigned to each pig $i$ without a phenotype. Group size equaled that at the start of the performance test as in the *full* scenario. The mean ADG and the average additive genetic relationship within pens were equivalent to those in the *full* scenario (Table 1).

### Estimation of genetic parameters

Variance components for ADG in each of the six scenarios were estimated using a classical genetic model (CGM), a standard indirect genetic model (IGM), and three IGM with weighting of indirect genetic effects: (1) with weighting *within* groups based on the relative time spent within that group ($IGM_{twg}$), (2) with weighting among groups based on relative space allowance ($IGM_{sbg}$), and, (3) with weighting both within and among groups ($IGM_{twsbg}$), combining $IGM_{twg}$ and $IGM_{sbg}$. The models and the concepts of relative time spent within the group and relative space allowance among groups are described in the following.

### The CGM was defined as

$${\mathbf{Y}} = {\mathbf{Xb}} + {\mathbf{Z}}_{{\mathbf{D}}} {\mathbf{a}}_{{\mathbf{D}}} + {\mathbf{W}}_{{\mathbf{l}}} {\mathbf{l}} + {\mathbf{W}}_{{\mathbf{g}}} {\mathbf{g}} + {\mathbf{e}},$$

where ${\mathbf{Y}}$ is the vector of $ADG$ during the performance test, and vector ${\mathbf{b}}$ includes regression coefficients on covariates and fixed effect estimates. Covariates were weight, age, and age squared of the pigs at the start of the performance test, as well as the average proportion of time that pigs spent with each other within each group, and the relative space allowance per group, $sbg_{grp}$ (as defined below). Fixed effects included the effect of the sex of the individual (boar or gilt) and the contemporary group, defined as pigs reared in pens within the same farm, barn, and batch based on the final test date. Each contemporary group included a minimum of four pens. Contemporary group was confounded with group size, i.e. group size was nested within contemporary group, except in scenarios *noPhen* and *testdays*, for which an additional fixed effect of group size was included. Vectors ${\mathbf{a}}_{{\mathbf{D}}}$, ${\mathbf{l}}$, and ${\mathbf{g}}$ represent random direct additive genetic effects, random litter effects, and random group effects, respectively, and ${\mathbf{e}}$ is a vector of residuals. Matrices ${\mathbf{X}}$, ${\mathbf{Z}}_{{\mathbf{D}}}$, ${\mathbf{W}}_{{\mathbf{l}}}$, and ${\mathbf{W}}_{{\mathbf{g}}}$ are incidence matrices. Assumptions for the distribution of random effects were:

$$\left[ {\begin{array}{*{20}c} {{\mathbf{a}}_{{\mathbf{D}}} } \\ {\begin{array}{*{20}c} {\mathbf{l}} \\ {\begin{array}{*{20}c} {\mathbf{g}} \\ {\mathbf{e}} \\ \end{array} } \\ \end{array} } \\ \end{array} } \right]\sim N\left( {\left[ {\begin{array}{*{20}c} {\mathbf{0}} \\ {\begin{array}{*{20}c} {\mathbf{0}} \\ {\begin{array}{*{20}c} {\mathbf{0}} \\ {\mathbf{0}} \\ \end{array} } \\ \end{array} } \\ \end{array} } \right],\left[ {\begin{array}{*{20}c} {{\mathbf{A}}\sigma_{{{\text{A}}_{\text{D}} }}^{2} } & 0 & 0 & 0 \\ 0 & {{\mathbf{I}}_{{\mathbf{l}}} \sigma_{\text{l}}^{2} } & 0 & 0 \\ 0 & 0 & {{\mathbf{I}}_{{\mathbf{g}}} \sigma_{\text{g}}^{2} } & 0 \\ 0 & 0 & 0 & {{\mathbf{I}}_{{\mathbf{e}}} \sigma_{\text{e}}^{2} } \\ \end{array} } \right]} \right),$$

where ${\mathbf{A}}$ is the additive relationship matrix, $\sigma_{{{\text{A}}_{\text{D}} }}^{2}$ is direct additive genetic variance, $\upsigma_{{\mathbf{l}}}^{2}$ is the variance of the birth litter, $\sigma_{\text{g}}^{2}$ is the variance of the group of pigs that were penned together during the performance test, $\sigma_{\text{e}}^{2}$ is the residual variance, and ${\mathbf{I}}_{{\mathbf{l}}}$, ${\mathbf{I}}_{{\mathbf{g}}}$, and ${\mathbf{I}}_{{\mathbf{e}}}$ are identity matrices of dimensions equal to the numbers of litters, groups, and observations on ADG, respectively. Fitting the group effect, ${\mathbf{g}}$, is equivalent to fitting correlated residuals among pen mates, assuming they are positively correlated [2].

The IGM was defined as:

$${\mathbf{Y}} = {\mathbf{Xb}} + {\mathbf{Z}}_{{\mathbf{D}}} {\mathbf{a}}_{{\mathbf{D}}} + {\mathbf{Z}}_{{\mathbf{I}}} {\mathbf{a}}_{{\mathbf{I}}} + {\mathbf{W}}_{{\mathbf{l}}} {\mathbf{l}} + {\mathbf{W}}_{{\mathbf{g}}} {\mathbf{g}} + {\mathbf{e}},$$

where ${\mathbf{a}}_{{\mathbf{I}}}$ is a vector of random indirect additive genetic effects, and the remaining model terms are as described for the CGM. Direct and indirect genetic effects were assumed to be distributed as

$$N\left( {0,{\mathbf{C}} \otimes {\mathbf{A}}} \right),$$

where

$${\mathbf{C}} = \left[ {\begin{array}{*{20}c} {\sigma_{{{\text{A}}_{\text{D}} }}^{2} } & {\sigma_{{{\text{A}}_{\text{DI}} }} } \\ {\sigma_{{{\text{A}}_{\text{DI}} }} } & {\sigma_{{{\text{A}}_{\text{I}} }}^{2} } \\ \end{array} } \right]$$

and $\otimes$ is the Kronecker product. Direct and indirect additive genetic effects were not correlated with the remaining random effects, which were distributed as:

$$\left[ {\begin{array}{*{20}c} {\mathbf{l}} \\ {\mathbf{g}} \\ {\mathbf{e}} \\ \end{array} } \right]\sim N\left( {\left[ {\begin{array}{*{20}c} {\mathbf{0}} \\ {\mathbf{0}} \\ {\mathbf{0}} \\ \end{array} } \right],\left[ {\begin{array}{*{20}c} {{\mathbf{I}}_{{\mathbf{l}}} \sigma_{\text{l}}^{2} } & 0 & 0 \\ 0 & {{\mathbf{I}}_{{\mathbf{g}}} \sigma_{\text{g}}^{2} } & 0 \\ 0 & 0 & {{\mathbf{I}}_{{\mathbf{e}}} \sigma_{\text{e}}^{2} } \\ \end{array} } \right]} \right).$$

Matrix ${\mathbf{Z}}_{{\mathbf{I}}}$ is an incidence matrix linking phenotypic values of individuals to the indirect genetic effects of group mates, with the elements, $s$, defined as: $s_{ii} = 0$ and $s_{ij} = 0$ when $i$ and $j$ are in different groups, whereas for $i$ and $j$ in the same group and $i \ne j$: $s_{ij} = 1$ for the IGM, and $s_{ij} = twg_{ij} /sbg_{grp} /twsbg_{ij}$ for the $IGM_{twg}$, $IGM_{sbg}$, and $IGM_{twsbg}$, respectively. $twg_{ij}$ is the proportion of time that each pig spends with other pigs within that group and $sbg_{grp}$. is the relative space allowance among groups:

$$twg_{ij} = \frac{{Ntestdays_{j} }}{{Ntestdays_{g} }},$$

where $Ntestdays_{j}$ is the number of days that a group mate, $j$, of pig $i$, spent in the group from the start of the test until it left the group or finished the performance test, and $Ntestdays_{g}$ is the number of days from start to finish of the performance test. When a group mate, $j$, stayed in the group throughout the performance test, then $twg_{ij} = 1$, whereas $0 < twg_{ij} < 1$ when a group mate left the group before the end of the performance test. This implies that a group mate that left the group prematurely had a smaller effect on the focal individual, than does a group mate that stayed in the group throughout the performance test. $sbg_{grp} = \frac{{\overline{{m_{g}^{2} /n_{end,g} }} }}{{m_{g}^{2} /n_{end,g} }}$, where $m_{g}^{2}$ is the square meters in the pen of group *g*, $n_{end,g}$ is the group size at the end of the performance test for group $g$, $m_{g}^{2} /n_{end,g}$ is the space allowance within the group, and $\overline{{m_{g}^{2} /n_{end,g} }}$ is the average space allowance across all groups within the data. When the space allowance within a group equals the average space allowance within the data, then $sbg_{grp} = 1$, whereas while $sbg_{grp} > 1$ when the space allowance within a group is smaller than the average value, and $sbg_{grp} < 1$ when the space allowance within a group is larger than the average value. This implies that indirect genetic effects increase for groups with a relatively small space allowance and decrease for groups with a relatively large space allowance. $twsbg_{ij}$ is the combined effect of $twg_{ij}$ and $sbg_{grp}$: $twsbg_{ij} = twg_{ij} *sbg_{grp}$. Both fixed regressions on $twg_{grp}$ and $sbg_{grp}$ are included in each model, because expectations must be zero, and if the means change as a result of covariates, then these covariates must be accounted for to ensure that expectations are zero. Both fixed regression coefficients on $twg_{grp}$ and $sbg_{grp}$ were significantly different from zero, but the average effect of $twsbg_{ij}$ per group was not, and thus was not included in the models. In the $IGM_{twg}$, $IGM_{sbg}$, and $IGM_{twsbg}$ indirect genetic effects are modeled as random regressions on either $twg_{ij}$, $sbg_{grp}$ or a combination of both. The genetic parameters (variance components) as well as estimated breeding values from the IGM models with weighting (random regression) are, therefore, functions of $twg_{ij}$, $sbg_{grp}$, and $twsbg_{ij}$. In the results, they are presented at the average value of these covariates.

Parameters in the models were estimated using average information residual maximum likelihood (AIREML) and genetic effects were best linear unbiased predictors (BLUP), which were in both cases implemented in the DMU software [14].

The phenotypic variance $\left( {\sigma_{P}^{2} } \right)$ for the IGM, $IGM_{twg}$, $IGM_{sbg}$, and $IGM_{twsbg}$ was calculated as proposed by Bergsma et al. [2] and modified by Ragab et al. [15]:

$$\sigma_{P}^{2} = \sigma_{{{\text{A}}_{\text{D}} }}^{2} + \sigma_{g}^{2} + \sigma_{l}^{2} + \sigma_{\text{e}}^{2} + c^{2} \left( {n - 1} \right)\sigma_{{{\text{A}}_{\text{I}} }}^{2} + c\left( {n - 1} \right)r\left[ {2\sigma_{{{\text{A}}_{\text{DI}} }} + c\left( {n - 2} \right)\sigma_{{{\text{A}}_{\text{I}} }}^{2} } \right],$$

where $\sigma_{{{\text{A}}_{\text{D}} }}^{2}$, $\sigma_{group}^{2}$, $\sigma_{litter}^{2}$, $\sigma_{\text{e}}^{2}$, $\sigma_{{{\text{A}}_{\text{I}} }}^{2}$, $\sigma_{{{\text{A}}_{\text{DI}} }}$, $n$, and $r$ are the direct additive genetic variance, the environmental variances of group and litter, the residual variance, the indirect additive genetic variance, the direct–indirect additive genetic covariance, the average group size (Table 1), and the average additive genetic relatedness among group mates (Table 1), respectively. The average weighing factor of indirect genetic effects $c$ was equal to 1, $\overline{{twg_{ij} }}$ (varying from 0.987 in *full* to 0.992 in *noPhenGrp15*), $\overline{{sbg_{grp} }}$ (varying from 1.011 in *full* to 1.025 in *noPhenGrp*), and $\overline{{twsbg_{ij} }}$ (varying from 0.999 in *full* to 1.017 in *noPhenGrp15*) for the IGM, $IGM_{twg}$, $IGM_{sbg}$, and $IGM_{twsbg}$, respectively (Table 1). For the IGM, the total heritable variance $\left( {\sigma_{TGE}^{2} } \right)$ of the total genetic effect (TGE) was calculated as proposed by Bijma et al. [16] and the total heritability $\left( {T^{2} } \right)$ was calculated as proposed by Bergsma et al. [2] and modified by Ragab et al. [15]:

$$\sigma_{TGE}^{2} = \sigma_{{{\text{A}}_{\text{D}} }}^{2} + 2c\left( {n - 1} \right)\sigma_{{{\text{A}}_{\text{DI}} }} + c^{2} \left( {n - 1} \right)^{2} \sigma_{{{\text{A}}_{\text{I}} }}^{2} ,$$

$$T^{2} = \sigma_{TGE}^{2} /\sigma_{P}^{2} .$$

It follows that $\sigma_{TGE}^{2}$ depends strongly on $n$. In fact, its magnitude can exceed that of $\sigma_{P}^{2}$, in which case $T^{2}$ > 1, and if $n \to \infty$ then $T^{2} \to \infty$. In this study, $n$ was equal to the average group size in each scenario.

Standard errors (SE) on estimates of direct heritability ($h_{D}^{2}$), $T^{2}$, and the genetic correlation between the direct and indirect genetic effects, $r_{{g_{DI} }}$, were calculated using equations that were derived by Bijma [17]:

$$\begin{aligned} & SE_{{h_{D}^{2} }} = SE_{{\sigma_{{{\text{A}}_{\text{D}} }}^{2} }} /\sigma_{P}^{2} ,SE_{{T^{2} }} = SE_{{\sigma_{TGE}^{2} }} /\sigma_{P}^{2} ,\;{\text{and}}\\ & SE_{{r_{{g_{DI} }} }} \approx \sqrt {\frac{{SE_{{\sigma_{{{\text{A}}_{\text{DI}} }} }}^{2} }}{{\sigma_{{{\text{A}}_{\text{D}} }}^{2} c^{2} \sigma_{{{\text{A}}_{\text{I}} }}^{2} }} + \frac{{c^{2} \sigma_{{{\text{A}}_{\text{DI}} }}^{2} Var\left( {\sigma_{{{\text{A}}_{\text{D}} }} \times \sigma_{{{\text{A}}_{\text{I}} }} } \right)}}{{\sigma_{{{\text{A}}_{\text{D}} }}^{4} c^{4} \sigma_{{{\text{A}}_{\text{I}} }}^{4} }}} ,\\ \end{aligned}$$

where $SE_{{\sigma_{TGE}^{2} }} = \frac{1}{r}\sqrt {\frac{2}{N - 1}\left[ {\sigma_{f}^{4} + \frac{{2\sigma_{f}^{2} \sigma_{\text{e}}^{2} }}{m} + \frac{{\sigma_{\text{e}}^{4} }}{{m\left( {m - 1} \right)}}} \right]}$, $\sigma_{f}^{2} = r\sigma_{TGE}^{2}$ is the between-family variance, $N$ is the number of families (litters in the data), $m$ is the family size (calculated as the total number of individuals in the dataset divided by the number of families/litters in the dataset), and $$\begin{aligned} {\text{Var}}\left( {\sigma _{{{\text{A}}_{{\text{D}}} }} \times \sigma _{{{\text{A}}_{{\text{I}}} }} } \right) & \approx \left[ {\frac{{\left( {SE_{{\sigma _{{{\text{A}}_{{\text{D}}} }}^{2} }} } \right)^{2} }}{{4\sigma _{{{\text{A}}_{{\text{D}}} }}^{2} }} + \sigma _{{{\text{A}}_{{\text{D}}} }}^{2} } \right] \times \left[ {\frac{{\left( {SE_{{\sigma _{{{\text{A}}_{{\text{I}}} }}^{2} }} } \right)^{2} }}{{4c^{2} \sigma _{{{\text{A}}_{{\text{I}}} }}^{2} }} + c^{2} \sigma _{{{\text{A}}_{{\text{I}}} }}^{2} } \right] \\ & \quad - \sigma _{{{\text{A}}_{{\text{D}}} }}^{2} c^{2} \sigma _{{{\text{A}}_{{\text{I}}} }}^{2} . \\ \end{aligned}$$

### Predictive ability and bias of predicted breeding values

The predictive ability and bias of predicted breeding values were calculated for the six scenarios by dividing the datasets described in Table 1 into training and validation datasets, with a cut-off date on January 1, 2017. Thus, the validation datasets included all pigs in groups that finished the performance test between January 1 and May 31, 2017, but their phenotypes were omitted. A 5-month period not only ensures a relatively large number of groups in the validation dataset, but also a relatively large number of sires with offspring and hence relatively high accuracies for both the training and validation datasets. Summary statistics for the validation datasets are in Table 2. For the six scenarios, the validation datasets differed with respect to the proportion of pigs without phenotypes (0% for *noPhen* and *predPhen* to 3.5% for *full*). Likewise, the proportion of groups that included pigs without phenotypes ranged from 0 (*noPhen* and *predPhen*) to 28.6% (*full*). One hundred and seventeen sires were represented with offspring in the validation data, but only 49 of them had offspring in the training dataset as well.

**Table 2:** Summary statistics of validation datasets in the reference scenario *full* and in the five alternative scenarios

	*full*	*noPhen*	*Testdays*	*noPhenGrp*	*noPhenGrp15*	*predPhen*
Number of records	10,589	10,217	10,542	7489	10,105	10,589
Number of records on offspring of sires also represented in training data	4954	4354	4492	3208	4332	4954
Number of groups	928	927	928	663	885	928
Group size; mean (min–max)	11.9 (8–15)	11.5 (5–15)	11.8 (6–15)	11.8 (8–15)	11.9 (8–15)	11.9 (8–15)
% pigs without phenotypes	3.5	0	3.0	0	2.5	0
% groups including pigs without phenotypes	28.6	0	25.3	0	25.1	0
Additive genetic relationship within pens; mean (min–max)	0.13 (0.07–0.43)	0.13 (0.07–0.43)	0.13 (0.07–0.43)	0.13 (0.07–0.43)	0.13 (0.07–0.43)	0.13 (0.07–0.43)
ADG; mean g/d ± sd (min–max)	1049 ± 122 (411–1698)	1049 ± 139 (411–1698)	1049 ± 121 (411–1698)	1051 ± 128 (561–1698)	1050 ± 129 (411–1698)	1048 ± 120 (411–1698)

Alternative scenarios were: *noPhen* (individually culled pigs or pigs without phenotypes were omitted), *testdays* (pigs culled within the first 28% of test days were omitted), *noPhenGrp* (complete groups with culled pigs or pigs with missing phenotypes omitted), *noPhenGrp15* (groups with more than 15% culled pigs or missing phenotypes were omitted), and *predPhen* (predicted breeding values assigned to pigs without phenotypes including culled pigs)

To further validate robustness of the results, we also defined validation datasets with two alternative cut-off points, i.e. on July 1, 2016, and January 1, 2016. These yielded similar results as the original validation datasets and are, therefore, not reported.

Predictive ability was defined as the Pearson correlation between predicted breeding values and corrected phenotypes (see below) in the validation dataset. Three alternative predicted breeding values were calculated: $a_{Di} = ebv_{D,i}$; the estimated direct breeding value of pig $i$.

$a_{Igrp} = \mathop \sum \nolimits_{j}^{n - 1} \left( {ebv_{I,j} \times s_{j} } \right)$; the sum of the estimated indirect breeding values of the group mates of a pig, $j$ = 1, 2, …, $n$, where $n$ is the group size, and $s_{j}$ is the weighing factor for the group mate.

$a_{Di,Igrp} = ebv_{D,i} + \mathop \sum \nolimits_{j}^{n - 1} \left( {ebv_{I,j} \times s_{j} } \right)$; the sum of the estimated direct breeding value of the pig and the estimated indirect breeding values of its group mates, $j$ = 1, 2, …, $n$.

The corrected phenotypes of each pig ($y_{Ci}$) were based on the model with the lowest AIC [18] applied to the *full* scenario. The AIC was used to compare models within a scenario as the fixed parts of the models were equivalent. The IGM, $IGM_{twg}$ and $IGM_{sbg}$ had the lowest AIC with a relative likelihood of 19 compared to that of the CGM and of 2 compared to that of $IGM_{twsbg}$. Thus, with IGM being the simplest model of the three with the lowest AIC, the corrected phenotypes were calculated based on the IGM as: the sum of all predicted random effects, including direct and indirect genetic, environmental random effects (litter, $\hat{l}_{i}$, and group, $\hat{g}_{i}$), and residuals, $\hat{e}_{i}$ (i.e., corrected phenotypes were equal to the difference between phenotypes and estimated fixed effects): $y_{c,i} = ebv_{D,i} + \mathop \sum \nolimits_{j}^{n - 1} \left( {ebv_{I,j} \times s_{j} } \right) + l_{i} + g_{i} + e_{i} .$

The Hotelling–Williams t-test [19] was applied to evaluate differences in predictive ability between models within each scenario. Differences were considered significant if the P-value of the test was less than 1%. To evaluate differences in predictive ability between scenarios, a two-tailed test of significant differences between independent correlations was applied, namely a Z-test based on a Fisher r-to-z transformation [20]. We chose this test because, although the datasets were not entirely independent, the data differed between scenarios.

Bias of predicted breeding values was calculated as the regression of breeding values post-phenotyping, $a_{post}$ (either $a_{Di}$ or $a_{Di,Igrp}$) for animals in the validation dataset, on the corresponding predicted breeding values (from the same model and scenario) prior to phenotyping ($a_{prior}$), also for animals in the validation dataset. Thus, $a_{post} = \beta_{0} + \beta_{1} \times a_{prior}$, where the extent of the deviation of $\beta_{1}$ from 1 reflects the amount of bias in the predicted breeding values.

## Results

### Genetic parameters

Table 3 presents estimates of variance components and of genetic parameters for several combinations of data editing procedures and models. The editing procedure did not have a significant effect on the estimated direct genetic variance, which ranged from 2437 (*noPhenGrp*) to 2675 (g/day)^2^ (*full*) for the CGM. Likewise, the editing procedure did not have a significant effect on the estimated indirect genetic variance, which ranged from 9.9 (*predPhen*) to 13.3 (g/day)^2^ (*noPhen*) for the IGM. The estimate of direct heritability ($h_{D}^{2}$) ranged from 0.23 (*noPhenGrp*) to 0.25 (*predPhen*), regardless of the model. When the IGM was used, the total genetic variance was larger than the direct genetic variance for all scenarios, thus, the estimates of $T^{2}$ were between 34 (*testdays* and *predPhen*) and 42% (*noPhen* and *noPhenGrp*) higher than the estimates of $h_{D}^{2}$ for the IGM. The genetic covariance between the direct and indirect genetic effects was negative (unfavorable) regardless of the editing procedure, but it was only significantly different from 0 for *predPhen* ($IGM_{twsbg}$). The genetic covariance was smallest for *noPhenGrp* (− 2.5) and largest (− 26.1 and − 29.7) for *predPhen* ($IGM_{twg}$ and $IGM_{twsbg}$). In the remaining scenarios, including *full*, the genetic covariance had an intermediate estimate (from − 9.0 to − 15.8). The estimate of the genetic correlation between direct and indirect effects ($r_{{g_{DI} }}$), which varied between − 0.02 (*noPhenGrp*) and − 0.19 (*noPhen*), only differed significantly from 0 (− 0.16 and − 0.19 ± 0.04) for *predPhen* ($IGM_{twg}$ and $IGM_{twsbg}$).

**Table 3:** Estimates of variance components and genetic parameters $h_{D}^{2}$, $T^{2}$^a^, and $r_{{g_{DI} }}$ (standard errors) based on a classical genetic model (CGM) and four indirect genetic models, without (IGM) or with weighting of indirect genetic effects ($IGM_{twg}$, $IGM_{sbg}$, and $IGM_{twsbg}$), applied to six editing procedure scenarios^b^

Scenario	Model	$\sigma_{{A_{D} }}^{2}$	$\sigma_{{A_{DI} }}$	$\sigma_{{A_{I} }}^{2}$	$\sigma_{g}^{2}$	$\sigma_{l}^{2}$	$\sigma_{E}^{2}$	$\sigma_{TGE}^{2}$	$h_{D}^{2}$	$T^{2}$	$r_{{g_{DI} }}$
*full*	$CGM$	2675 (127)	–	–	1095 (28)	654 (27)	6542 (68)	–	0.24 (0.01)	–	–
	$IGM$	2667 (128)	− 9.5 (14.5)	10.3 (2.4)	1008 (36)	652 (27)	6531 (69)	3619 (94)	0.24 (0.01)	0.33 (0.01)	− 0.06 (0.07)
	$IGM_{twg}$	2666 (128)	− 11.2 (14.6)	10.5 (2.4)	1010 (37)	652 (27)	6530 (69)	3574 (95)	0.24 (0.01)	0.32 (0.01)	− 0.07 (0.06)
	$IGM_{sbg}$	2667 (128)	− 9.5 (14.5)	10.3 (2.4)	1008 (36)	652 (27)	6531 (69)	3644 (93)	0.24 (0.01)	0.33 (0.01)	− 0.06 (0.07)
	$IGM_{twsbg}$	2664 (128)	− 11.3 (13.9)	8.7 (2.1)	1022 (36)	653 (27)	6531 (69)	3396 (93)	0.24 (0.01)	0.31 (0.01)	− 0.07 (0.06)
*noPhen*	$CGM$	2675 (127)	–	–	1095 (28)	654 (27)	6542 (68)	–	0.24 (0.01)	–	–
	$IGM$	2664 (128)	− 12.5 (15.4)	13.3 (2.7)	986 (36)	652 (27)	6528 (69)	3780 (96)	0.24 (0.01)	0.34 (0.01)	− 0.07 (0.05)
*testdays*	$CGM$	2675 (127)	–	–	1095 (28)	654 (27)	6542 (68)	–	0.24 (0.01)	–	–
	$IGM$	2665 (128)	− 11.6 (14.6)	10.4 (2.4)	1010 (37)	652 (27)	6530 (69)	3572 (94)	0.24 (0.01)	0.32 (0.01)	− 0.07 (0.06)
	$IGM_{twg}$	2665 (128)	− 11.5 (14.7)	10.5 (2.5)	1010 (37)	652 (27)	6530 (69)	3570 (95)	0.24 (0.01)	0.32 (0.01)	− 0.07 (0.06)
	$IGM_{sbg}$	2664 (128)	− 11.7 (13.9)	8.6 (2.1)	1022 (37)	653 (27)	6531 (69)	3387 (95)	0.24 (0.01)	0.31 (0.01)	− 0.08 (0.05)
	$IGM_{twsbg}$	2664 (128)	− 11.5 (13.9)	8.7 (2.1)	1021 (37)	653 (27)	6531 (69)	3392 (95)	0.24 (0.01)	0.31 (0.01)	− 0.08 (0.05)
*noPhenGrp*	$CGM$	2437 (137)	–	–	1007 (32)	688 (34)	6273 (75)	–	0.23 (0.01)	–	–
	$IGM$	2431 (138)	− 2.5 (16.4)	10.0 (2.9)	916 (44)	686 (34)	6269 (76)	3464 (124)	0.23 (0.01)	0.33 (0.01)	− 0.02 (0.10)
*noPhenGrp15*	$CGM$	2670 (130)	–	–	1090 (28)	658 (28)	6506 (69)	–	0.24 (0.01)	–	–
	$IGM$	2660 (130)	− 10.5 (15.0)	11.0 (2.6)	998 (38)	656 (28)	6495 (70)	3679 (99)	0.24 (0.01)	0.33 (0.01)	− 0.06 (0.06)
	$IGM_{twg}$	2660 (130)	− 11.1 (15.1)	11.1 (2.6)	999 (38)	656 (28)	6494 (70)	3657 (99)	0.24 (0.01)	0.33 (0.01)	− 0.07 (0.06)
	$IGM_{sbg}$	2660 (130)	− 10.4 (14.2)	8.9 (2.2)	1011 (37)	656 (28)	6496 (70)	3482 (95)	0.24 (0.01)	0.32 (0.01)	− 0.07 (0.06)
	$IGM_{twsbg}$	2659 (130)	− 10.8 (14.3)	9.0 (2.2)	1011 (37)	656 (28)	6495 (70)	3477 (95)	0.24 (0.01)	0.32 (0.01)	− 0.07 (0.06)
*predPhen*	$CGM$	2643 (122)	–	–	1092 (26)	654 (26)	6176 (65)	–	0.25 (0.01)	–	–
	$IGM$	2635 (123)	− 9.0 (14.0)	9.9 (2.3)	1009 (35)	652 (26)	6166 (65)	3550 (90)	0.25 (0.01)	0.33 (0.01)	− 0.06 (0.07)
	$IGM_{twg}$	2627 (122)	− 26.1 (14.2)	10.6 (2.4)	1024 (35)	651 (26)	6152 (66)	3236 (88)	0.25 (0.01)	0.30 (0.01)	− 0.16 (0.04)
	$IGM_{sbg}$	2629 (122)	− 15.8 (13.5)	8.8 (2.1)	1023 (37)	652 (26)	6161 (65)	3296 (95)	0.25 (0.01)	0.31 (0.01)	− 0.10 (0.05)
	$IGM_{twsbg}$	2626 (122)	− 29.7 (13.6)	9.5 (2.2)	1034 (37)	651 (26)	6149 (65)	3065 (95)	0.25 (0.01)	0.29 (0.01)	− 0.19 (0.04)

^a^Calculated at $n$ equal to the average group size within each scenario

^b^The *full* scenario with no editing based on culled pigs or pigs without phenotypes and five alternative scenarios: *noPhen* (individually culled pigs or pigs without phenotypes were omitted), *testdays* (pigs culled within the first 28% of test days were omitted), *noPhenGrp* (complete groups with culled pigs or pigs with missing phenotypes omitted), *noPhenGrp15* (groups with more than 15% culled pigs or missing phenotypes were omitted), and *predPhen* (predicted breeding values assigned to pigs without phenotypes including culled pigs)

Model choice did not affect the estimate of the direct genetic variance. For example, for the *full* scenario, the estimate of the direct genetic variance did not differ significantly between either of the IGM models and the CGM. The estimate of the indirect genetic variance tended to be larger for $IGM_{twg}$ than for IGM (between 1 and 7%), and smaller for $IGM_{sbg}$ (except for the *full* scenario) and $IGM_{twsbg}$ (between − 4 and 20%). The estimate of the total genetic variance available for selection was between 16 and 42% larger than the estimate of the direct genetic variance, with the difference being largest for the IGM applied to *noPhenGrp* (42%) and *noPhen* (41%). Thus, the effect of including indirect genetic effects on the estimated total genetic variance was largest when the editing procedure involved omission of either all culled individuals or all groups with culled animals. Within each scenario, the estimate of the total genetic variance tended to be smaller when indirect genetic effects were weighted (up to 9, 7 and 14% for the *predPhen* scenario for $IGM_{twg}$, $IGM_{sbg}$, and $IGM_{twsbg}$, respectively) than when they were not (IGM).

Within each scenario, estimates of $h_{D}^{2}$, $T^{2}$, and $r_{{g_{DI} }}$ did not differ significantly among models. Estimates of $r_{{g_{DI} }}$ tended to be more negative for the IGM with weighting of indirect genetic effects (between − 0.10 and − 0.19) than for the IGM without weighting (− 0.06) but only in the *predPhen* scenario.

For the IGM models with weighting (random regression), $\sigma_{TGE}^{2}$ is a co-variance function with covariables $twg_{ij}$, $sbg_{grp}$, or $twsbg_{ij}$. Therefore, $T^{2}$ depends on the level of the covariables and in the extreme cases in the *full* scenario, $T^{2}$ varied between 0.24 for $IGM_{twsbg}$ at the minimum value of $twsbg_{ij}$ and 0.37 for $IGM_{sbg}$ at the maximum value of $sbg_{grp}$. The genetic correlation between TGE evaluated at different values for the covariables deviate slightly from 1 and in the most extreme case ($IGM_{twsbg}$) the genetic correlation between TGE evaluated at minimum and maximum value for $twsbg_{ij}$ was 0.83.

There were significant group and litter effects and, although the estimate of the group variance was 5 to 10% smaller for the IGM than for the CGM, this difference was not statistically significantly different from zero, regardless of weighting of indirect genetic effects or editing procedure. Estimates of litter and residual variances also did not differ significantly between models.

### Predictive ability

The predictive ability of the predicted direct breeding values of individual pigs, $a_{Di}$, the sum of the predicted indirect breeding values of group mates, $a_{Igrp}$, and the combination of the two, $a_{Di,Igrp}$, were reflected in their correlations with corrected phenotypes in the validation data (Table 4). The predictive abilities of $a_{Di}$ were higher for *noPhenGrp* and *noPhenGrp15* than for the other editing procedure scenarios (between 1 and 3% higher). The predictive ability of $a_{Di,Igrp}$ from the IGM was approximately 1% higher than that of $a_{Di}$ for all scenarios. For all scenarios, except *noPhen*, the differences in predictive abilities were significantly different from zero. The predictive ability of $a_{Di,Igrp}$ was lowest for scenario *noPhen* and highest for *noPhenGrp* (4% higher than for *noPhen*). The predictive ability of $a_{Igrp}$ was highest, by between 8 and 46%, for the scenario *full*, compared to the other scenarios.

**Table 4:** Predictive abilities^a^ of the classical genetic model (CGM) and four indirect genetic models without (IGM) or with weighting of indirect genetic effects ($IGM_{twg}$, $IGM_{sbg}$, and $IGM_{twsbg}$) for six editing procedure scenarios^b^

Scenario	Model	$cor\left( {Y_{c,i} ,a_{Di} } \right)$	$cor\left( {Y_{c,i} ,a_{Igrp} } \right)$	$cor\left( {Y_{c,i} ,a_{Di,Igrp} } \right)$
*full*	${\text{CGM}}$	0.248^a^	–	–
	${\text{IGM}}$	0.248^a^	0.038^a^	0.251^a^
	$IGM_{twg}$	0.248^a^	0.029^b^	0.251^a^
	$IGM_{sbg}$	0.248^a^	0.032^c^	0.251^a^
	$IGM_{twsbg}$	0.248^a^	0.015^d^	0.250^b^
*noPhen*	${\text{CGM}}$	0.248^a^	–	–
	${\text{IGM}}$	0.248^a^	− 0.074	0.248
*testdays*	${\text{CGM}}$	0.248^a^	–	–
	${\text{IGM}}$	0.248^a^	0.034^a^	0.251^a^
	$IGM_{twg}$	0.248^a^	0.027^b^	0.251^a^
	$IGM_{sbg}$	0.248^a^	0.026^b^	0.251^a^
	$IGM_{twsbg}$	0.248^a^	0.013^c^	0.249^b^
*noPhenGrp*	${\text{CGM}}$	0.256^a^	–	
	${\text{IGM}}$	0.256^a^	0.026	0.258
*noPhenGrp15*	${\text{CGM}}$	0.250^a^	–	–
	${\text{IGM}}$	0.250^a^	0.028^a^	0.252^a^
	$IGM_{twg}$	0.250^a^	0.021^b^	0.252^a^
	$IGM_{sbg}$	0.250^a^	0.017^c^	0.251^b^
	$IGM_{twsbg}$	0.250^a^	0.007^d^	0.250^c^
*predPhen*	${\text{CGM}}$	0.249^a^	–	–
	${\text{IGM}}$	0.248^b^	0.038^a^	0.251^a^
	$IGM_{twg}$	0.249^a^	0.008^b^	0.250^b^
	$IGM_{sbg}$	0.250^c^	0.007^b^	0.250^b^
	$IGM_{twsbg}$	0.249^a^	− 0.005^c^	0.248^c^

Different letters indicate significant (P ≤ 1%) differences between models within the columns and scenarios

^a^Predictive abilities were calculated as the correlation between predicted breeding values and corrected phenotypes for: individual direct breeding values, $a_{Di}$, the sum of the indirect breeding values of the group mates, $a_{Igrp}$, and the combination of direct, individual breeding value and the sum of the indirect breeding values of the group mates,$a_{Di,Igrp}$

^b^The *full* scenario with no editing based on culled pigs or pigs without phenotypes and five alternative scenarios: *noPhen* (individually culled pigs or pigs without phenotypes were omitted), *testdays* (pigs culled within the first 28% of test days were omitted), *noPhenGrp* (complete groups with culled pigs or pigs with missing phenotypes omitted), *noPhenGrp15* (groups with more than 15% culled pigs or missing phenotypes were omitted), and *predPhen* (predicted breeding values assigned to pigs without phenotypes including culled pigs)

Within a scenario, the predictive ability of $a_{Di,Igrp}$ was always equal to or higher (up to 1% for *predPhen*) for the IGM than for the other models. Likewise, the predictive ability of $a_{Igrp}$ was always higher for the IGM than for the other models (between 19 and 153% for *full*), i.e. weighting indirect genetic effects based on relative time spent in the pen or space allowance in the model did not improve predictive ability.

### Bias of predicted breeding values

Bias of predicted breeding values was reflected by the extent to which the regression coefficient of predicted breeding values post-phenotyping on predicted breeding values prior to phenotyping deviated from 1 (Table 5). For the scenarios *full*, *noPhen*, and *testdays*, $a_{Di}$ was not biased regardless of the model, but for *predPhen*, $a_{Di}$ was biased when using the $IGM_{sbg}$ model. For the scenario *noPhenGrp15* and in particular for *noPhenGrp*, $a_{Di}$ was biased regardless of the model. Likewise, the predicted combined direct and sum of indirect breeding values, $a_{Di,Igrp}$, from model $IGM_{twg}$ was biased, except for the scenario *testdays*. Lastly, all IGM models resulted in biased $a_{Di,Igrp}$ in the scenarios *noPhenGrp* and *noPhenGrp15*, with the largest bias of $a_{Di,Igrp}$ observed for scenario *noPhenGrp* (1.100). For a given scenario, there was generally no significant difference in bias for $a_{Di,Igrp}$ between the IGM models with or without weighting of indirect genetic effects. Except for scenario *predPhen*, for which the bias of $a_{Di,Igrp}$ was significantly larger for model $IGM_{sbg}$ than for the other IGM models.

**Table 5:** Bias^a^ (standard errors in parentheses) of the classical genetic model (CGM) and four indirect genetic models without (IGM) or with weighting of indirect genetic effects ($IGM_{twg}$, $IGM_{sbg}$, and $IGM_{twsbg}$) for six editing procedure scenarios^b^

Scenario	Model	$a_{Di}$	$a_{Di,Igrp}$
*full*	${\text{CGM}}$	1.002^a^ (0.009)	–
	${\text{IGM}}$	1.005^a^ (0.009)	1.017^b^ (0.009)
	$IGM_{twg}$	1.005^a^ (0.009)	1.018^b^ (0.009)
	$IGM_{sbg}$	1.004^a^ (0.009)	1.019^b^ (0.009)
	$IGM_{twsbg}$	1.005^a^ (0.009)	1.016^b^ (0.009)
*noPhen*	${\text{CGM}}$	1.002^a^ (0.009)	–
	${\text{IGM}}$	0.999^a^ (0.009)	1.000^a^ (0.009)
*testdays*	${\text{CGM}}$	1.002^a^ (0.009)	–
	${\text{IGM}}$	1.005^a^ (0.009)	1.016^b^ (0.009)
	$IGM_{twg}$	1.005^a^ (0.009)	1.018^b^ (0.009)
	$IGM_{sbg}$	1.005^a^ (0.009)	1.016^b^ (0.009)
	$IGM_{twsbg}$	1.005^a^ (0.009)	1.016^b^ (0.009)
*noPhenGgrp*	${\text{CGM}}$	1.084^b^ (0.009)	–
	${\text{IGM}}$	1.084^b^ (0.009)	1.100^b^ (0.008)
*noPhenGrp15*	${\text{CGM}}$	1.021^b^ (0.009)	–
	${\text{IGM}}$	1.023^b^ (0.009)	1.035^b^ (0.009)
	$IGM_{twg}$	1.023^b^ (0.009)	1.035^b^ (0.009)
	$IGM_{sbg}$	1.023^b^ (0.009)	1.034^b^ (0.009)
	$IGM_{twsbg}$	1.023^b^ (0.009)	1.033^b^ (0.009)
*predPhen*	${\text{CGM}}$	0.997^a^ (0.009)	–
	${\text{IGM}}$	0.999^a^ (0.009)	1.012^a^ (0.009)
	$IGM_{twg}$	1.000^a^ (0.009)	1.014^a^ (0.009)
	$IGM_{sbg}$	1.023^b^ (0.009)	1.033^b^ (0.009)
	$IGM_{twsbg}$	1.000^a^ (0.009)	1.012^a^ (0.009)

Different letters indicate significant (P ≤ 5%) bias within model and scenario, where $H_{0} :\beta = 1$

^a^Bias was defined as the extent to which the regression of the predicted breeding values post-phenotyping on the equivalent breeding values prior to phenotyping differed from 1. Breeding values were either direct breeding values, $a_{Di}$, or the combination of the direct breeding value and the sum of the indirect breeding values of the group mates, $a_{Di,Igrp}$

^b^The *full* scenario with no editing based on culled pigs or pigs without phenotypes and five alternative scenarios: *noPhen* (individually culled pigs or pigs without phenotypes were omitted), *testdays* (pigs culled within the first 28% of test days were omitted), *noPhenGrp* (complete groups with culled pigs or pigs with missing phenotypes omitted), *noPhenGrp15* (groups with more than 15% culled pigs or missing phenotypes were omitted), and *predPhen* (predicted breeding values assigned to pigs without phenotypes including culled pigs)

## Discussion

We showed that, in general, neither editing procedures related to culled animals and missing phenotypes, nor accounting for culled animals in the IGM, affect estimates of genetic parameters significantly. We also showed that editing procedures that remove culled animals or pigs with missing phenotypes but not their group mates, tend to reduce the predictive ability of estimates of indirect genetic effects ($cor\left( {Y_{c,i} ,a_{Igrp} } \right)$). In contrast, editing procedures, which remove full groups that include culled pigs or pigs with missing phenotypes, improve the predictive ability of estimates of direct genetic effects ($cor\left( {Y_{c,i} ,a_{Di} } \right)$), but increase their bias. Moreover, we found that accounting for culled animals in the model reduced the predictive ability of estimates of indirect genetic effects and sometimes increased bias of the predicted breeding values.

### Editing procedure scenarios

Our results showed that genetic parameters were not affected by editing procedures related to culled animals, and this indicates that it is not necessary to omit individual animals or whole groups from data if culled animals are present when estimating genetic parameters (at least for the relatively low frequency of culling in the present dataset).

Most other studies that estimated genetic parameters based on IGM did not report frequencies of culled animals or how these were handled e.g. [2, 4, 21, 22]. Arango et al. [6] omitted individual records that were outside a perceived acceptable phenotypic range of ADG, which is somewhat similar to the *noPhen* scenario. Bunter et al. [7] excluded entire groups that included missing individual(s), which is similar to the *noPhenGrp* scenario. Based on our results, the editing procedures used in these studies were not necessary when estimating genetic parameters, which is surprising because culling of animals is likely not random and possibly associated with indirect genetic effects. For example, pigs that are culled due to illness likely have a low ADG and pigs that are sold or without phenotypes likely have high ADG and a high parent average breeding value for ADG. Non-random culling of animals can bias estimates of genetic parameters [8–10], whereas random culling of individuals was shown to not affect estimates of genetic parameters, using simulation [23]. This suggests that culling of animals in our study was close to random or at least not associated with indirect genetic effects. Alternatively, it is possible that different culling reasons have opposite effects on indirect genetic effects, and that their occurrence cancelled each other out in our study. The effect of different culling reasons on indirect genetic effects was not studied here, because the frequencies of culling reasons were assumed to be too low and unevenly distributed for the detection of any impact.

The *noPhen* and *testdays* scenarios were also expected to affect estimates of genetic parameters, because culling of animals reduces the group size of affected groups. An IGM with a dilution effect was previously suggested to account for a reduction in indirect genetic effects with increasing group size [21, 24]. In contrast, genetic parameter estimates from the *noPhen* and *testdays* scenarios indicate that group size did not affect indirect genetic effects. To confirm this finding, we tested different degrees of dilution, ranging from 0.1 to 1.0 (results not shown). And indeed, dilution neither improved model fit (AIC) nor changed genetic parameter estimates significantly, in agreement with previous studies [6, 25].

Our results also showed that omitting individuals or groups from data based on culled animals reduces the predictive ability of estimated breeding values, whereas omission of entire groups with culled animals (*noPhenGrp* and *noPhenGrp15*) introduced bias in predicted breeding values. This indicates that, if there is indeed an association between culled animals and indirect genetic effects for growth, then the IGE cannot be disentangled in the analysis based on the data on individuals that remained in the groups. Omission of entire groups may result in bias because of confounding of the fixed contemporary and random group effects, as well as between the random group and litter effects. In conclusion, individuals or groups should not be removed from the data for IGM analyses to account for culled animals or missing phenotypes.

### Models and genetic parameters

We identified significant indirect genetic effects for ADG, with the $T^{2}$ of ~ 0.33 being higher than the $h_{D}^{2}$ of 0.24. We also found a negative $r_{{g_{DI} }}$ (− 0.07), but this was non-significantly different from zero, and hence neither indicating competition nor cooperation. The estimates of indirect genetic variances in our study were small [e.g. 10.3 (g/day)^2^ for IGM in *full*] particularly so in comparison to the estimates of direct genetic variances. Such (relatively) low values have in the past been mistakenly interpreted as unimportant [26], but their contribution to the total genetic variance is in fact substantial, as indicated by the $T^{2}$ because this depends on the group size [2]. Standard errors of the estimates (± 2.4) also indicate that they are in fact present and varying the prior of the indirect genetic variance in the IGM between 10 and 50 by 10 did not affect convergence or results, indicating a global maximum of the likelihood function.

Several previous studies have also identified significant indirect genetic effects for ADG in pigs, which fall mostly within the same range as in our study. Chen et al. [22] reported a $h_{D}^{2}$ of 0.20 and an indirect heritability of 0.001, which are similar to our estimates, but they found a positive $r_{{g_{DI} }}$ of 0.24 resulting in a much higher $T^{2}$ of 0.61, which contrasts with our results. However, comparing studies based on $T^{2}$ is difficult because $T^{2}$ depends on group size. For example, Chen et al. [22] had a group size of 15 compared to 11.6 in the *full* scenario in our study, which would also have contributed to the higher $T^{2}$. Bergsma et al. [27] found a $T^{2}$ of 0.34, which is similar to our results but their study was based on a smaller group size, i.e. 8.5. Duijvesteijn et al. [4] and Nielsen et al. [3] also reported genetic parameters for ADG in pigs similar to our results but the results of Duijvesteijn et al. [4] were based on a smaller group size (9.8). Canario et al. [21] found a lower $T^{2}$ (0.23), but this was probably due to both a much lower $h_{D}^{2}$ (0.13), as well as a smaller group size (8.5), and not due to smaller indirect genetic effects. Hence, our results confirm that there are indirect genetic effects for growth in pigs.

Our results showed that indirect genetic effects are not significantly affected by weighting of indirect genetic effects based on the relative proportion of time spent in the pen. This indicates that indirect interactions between pigs within a pen are not affected by the amount of time that the pigs have spent together when only a relatively small proportion of the pigs are removed (3.2% of the pigs across 24.8% of the groups). This implies that an aggressive pig has the same effect on the ADG of its group mates over the test period, regardless of whether it has been in the pen throughout the entire test period or it was culled before the end of the test. This may seem to disagree with results of ethological studies, which show that persisting aggression can affect ADG at the phenotypic level [28]. However, in our analyses, we also corrected for the mean change in ADG due to changes in the mean proportion of time spent together in the pen, which may be sufficient to explain the effects observed in ethological studies. The regression coefficient of the mean proportion of time spent together in the pen on ADG was estimated to be − 71 ± 31 g/day/%, which implies that each 1%-point increase in the average proportion of time spent together in the pen reduced the ADG by 71 g/day. Alternatively, the culled pigs may have been non-central to the social network within the group [29, 30]. If “outsider” pigs that barely interact with the other pigs are culled, they will not have much indirect genetic effect on their group mates regardless of being culled or not. In summary, estimates of indirect genetic effects are not enhanced by weighting based on either the relative proportion of time spent in the pen or based on the relative space allowance.

Our results also show that estimates of indirect genetic effects are not significantly affected by weighting based on the relative space allowance between pens ($IGM_{sbg}$), when only a relatively small proportion of the pigs are removed. This implies that space allowance is not important for indirect genetic effects and that these do not increase with decreasing space allowance. However, findings from ethological studies in pigs imply the opposite, i.e. that lower space allowance increases injuries and aggression [30, 31]. Similarly, in fish, aggression is known to increase with increasing space allowance or to have an optimum, at least within the range of space allowance typically practiced in commercial fish breeding [32, 33]. However, these effects observed in ethological studies may be fully explained by the model correction for the mean change in ADG as a result of differences in the relative space allowance. The regression coefficient of the relative space allowance on ADG was estimated to be 0.5 ± 14 g/day per unit increase in relative space allowance, which was not significantly different from zero. The lack of significance of weighting IGE based on the relative space allowance between pens may be explained by the heterogeneity in space allowance being too small to detect statistically significant differences. In addition, the results disagree with the results of Bunter et al. [7], who concluded that indirect genetic effects of sows on litter size decreased as space allowance increased irrespectively of maximum group size.

Our results were based on an evaluation of the genetic parameters at the mean level of the covariates $twg_{ij}$, $sbg_{grp}$, and $twsbg_{ij}$, which in all cases were close to 1. However, in the extreme case of the minimum level of the combined effect of the proportion of time spent in the group and the relative space allowance ($twsbg_{ij}$), the total genetic variance available for selection $T^{2}$ was reduced to the same level as $h_{D}^{2}$ (0.24), and the total genetic correlation between ADG evaluated at the minimum or maximum value of $twsbg_{ij}$ in $IGM_{twsbg}$ in the *full* scenario was 0.83. This means that the effect of selection may be lower than expected depending on the value of the covariables. If selection is aimed at an environment with a higher proportion of animals prematurely removed from groups and a higher space allowance per pig then realized selection response might be lower than expected.

### Models and predictive ability

The predictive ability of $a_{Di,Igrp}$ was 1% higher for the IGM than for the CGM ($a_{Di}$). This means that selection for ADG would be improved by using the IGM rather than the CGM, but it was not improved further by weighting indirect genetic effects. This indicates that it is not necessary to take the timing of the culling of animals or changes in space allowance due to culling of animals into account when evaluating the added value of indirect genetic effects. On the contrary, the predictive ability of the predicted indirect genetic effects ($a_{Igrp}$) decreased when the indirect genetic effects were weighted. Moreover, editing procedures involving omissions of culled animals or groups with culled animals from the data also reduced the predictive ability of the predicted indirect genetic effects. This suggests that editing procedures or weighting of indirect genetic effects based on culling of animals should not be done, regardless of whether they are based on the relative proportion of time in the pen or the relative space allowance between pens. In this study, we presented several methods to weight indirect genetic effects based on culled animals but these did not improve the results. However, it is possible that weighting of indirect genetic effects based on other factors that describe covariances among all pen mates more accurately will improve results. For example, social network analysis parameters, such as degree centrality or pairwise time spent fighting [34], or automatically recorded feeding behavior variables [35], such as Euclidian distance [15], could be applied as weighting factors. However, such data were not available in our study and are typically not available on a large scale in breeding programs. Weighting of indirect genetic effects based on relatedness may also be relevant [36, 37]. Alemu et al. [36] presented a model that takes differences in indirect genetic effects between relatives and unrelated individuals into account. However, even with a group structure that allows identification of all covariance components in this model, i.e. with variation in relatedness in group composition, the results of such a model may be biased if the differences in indirect genetic effects between related and unrelated animals are also affected by e.g. familiarity. Information on early-life experience may allow these effects to be disentangled [38], but such information was not available in our study.

The magnitude of the predictive abilities of $a_{Di}$ from the CGM and $a_{Di,Igrp}$ from the IGM in our study were similar to those found for gilts in Nielsen et al. [3], although their predictive abilities of predicted indirect genetic effects were much higher (between 0.067 and 0.165) than those found in our study (between − 0.074 and 0.038). Nielsen et al. [3] calculated predictive abilities only for complete groups, as suggested by Duijvesteijn et al. [4], thus we did the same, i.e. only for groups for which all phenotypes were known and no animals were culled. However, our *noPhenGrp* scenario is different to the situation in Nielsen et al. [3] because they did not omit individuals or groups when estimating the genetic variance. Their hypothesis was that the missing phenotypes may reduce accuracy of or bias the estimates of indirect genetic effects of pigs in affected groups [4, 9, 39]. Indeed, we found that the predictive abilities of indirect genetic effects were higher for complete groups than for all groups. For example, the predictive ability of $a_{Igrp}$ based on the IGM in the scenario *full* increased by 40% from 0.038 to 0.053. Except for the scenario *noPhen*, the predictive abilities of the predicted indirect genetic effects in the other scenarios similarly increased by 38 to 73%. The increase in predictive ability of the sum of direct and indirect genetic effects ($a_{Di,Igrp}$) of the IGM over that of the CGM was also 1 to 2 or 2.5% greater for full groups.

### Practical implications

Our results show that it is important to not omit individuals or groups based on culled animals during data editing when estimating indirect genetic effects, as this may compromise their predictive ability, as a reflection of accuracy of selection, and thereby genetic gain in the indirect genetic effects. The results also imply that the frequency of culled animals to the extent observed in this paper (~ 3% of individuals and ~ 25% of groups affected) should not be an issue when selecting on estimates of indirect genetic effects. In fact, animals that have been culled are important for the estimation of indirect genetic effects and must be included in analyses. Our results also show that weighting of indirect genetic effects of culled animals reduces the predictive ability of estimated indirect genetic effects. Instead, fixed regressions on the average relative proportion of time spent within the pen and the relative space allowance can account for the association of culled animals with ADG within groups. This stresses that it is important to take fixed effects that may otherwise be captured by the indirect genetic variance when modelling indirect genetic effects into account.

## Conclusions

Our results confirm that there are indirect genetic effects for growth in pigs, but indirect genetic effects of culled animals should not be removed through data editing, as this will reduce predictive ability and increase bias of estimated breeding values. In fact, animals that have been culled are important when estimating indirect genetic effects and must be included in analyses with IGM. Moreover, indirect genetic effects of culled animals should not be weighted based on proportion of time spent in the pen or relative space allowance as this will also reduce predictive ability. Instead, the effects of culled animals on indirect genetic effects should be accounted for by including fixed regressions of the mean proportion of time spent in the pen or relative space allowance on ADG in the model.

## Data Availability

The datasets analyzed during the current study are not publicly available because they are owned by SEGES P/S.
